# Multiple presence of prothrombotic risk factors in Croatian children with arterial ischemic stroke and transient ischemic attack

**DOI:** 10.3325/cmj.2013.54.346

**Published:** 2013-08

**Authors:** Jasna Leniček Krleža, Vlasta Đuranović, Ana Bronić, Desiree Coen Herak, Vlatka Mejaški-Bošnjak, Renata Zadro

**Affiliations:** 1 Department of Laboratory Diagnostics, Children's Hospital Zagreb, Zagreb, Croatia; 2 Department of Neuropediatrics, Children's Hospital Zagreb, Zagreb, Croatia; 3 Department of Laboratory Diagnostics, University Hospital Center “Sestre milosrdnice,” Hospital for Traumatology, Zagreb, Croatia; 4 Clinical Institute of Laboratory Diagnosis, University School of Medicine and University Hospital Center Zagreb, Zagreb, Croatia

## Abstract

**Aim:**

To determine the frequency of inherited and acquired prothrombotic risk factors in children with arterial ischemic stroke (AIS) and transient ischemic attacks (TIA) in Croatia.

**Methods:**

We investigated 14 prothrombotic risk factors using blood samples from 124 children with AIS or TIA and 42 healthy children. Prothrombotic risk factors were classified into five groups: natural coagulation inhibitors (antithrombin, protein C, protein S), blood coagulation factors (FV Leiden and FII 20210), homocysteine, lipid and lipoprotein profile (lipoprotein (a), triglycerides, total, high- and low-density lipoprotein), and antiphospholipid antibodies (lupus anticoagulant, anticardiolipin, and antiphosphatidylserine antibodies).

**Results:**

The most common prothrombotic risk factor was elevated lipoprotein (a), which was identified in about 31% of patients and in 24% of controls. Natural coagulation inhibitors were decreased in about 19% of patients, but not in controls. Pathological values of homocysteine, blood coagulation factor polymorphisms, and antiphospholipid antibodies were found in similar frequencies in all groups. Fourteen children with AIS and TIA (11.3%) and no children from the control group had three or more investigated risk factors.

**Conclusion:**

The presence of multiple prothrombotic risk factors in children with cerebrovascular disorder suggests that a combination of risk factors rather than individual risk factors could contribute to cerebrovascular disorders in children.

Stroke in children is a heterogeneous disorder, increasingly recognized as an important cause of childhood disability and lifelong morbidity (1). In up to one third of children with arterial ischemic stroke (AIS), preceding transient ischemic attacks (TIA) are present, although they are frequently not diagnosed (2).

The frequency of ischemic stroke in children may be greater than previously suggested, as reported in a recent study performed in a cohort of North Californian children, which found a two to four times higher frequency than previously estimated in the US (3). Recent estimates have suggested that ischemic stroke in children occurs at much higher rate soon after birth than later in childhood: in about 1 per 4000 live births in the US (4). In Croatia, the yearly incidence of AIS in children is 0.67 cases per 100 000 (5).

Although modern technology allows accurate definition of the presence and type of stroke, in up to one third of children the etiology still remains undetermined (6). Conditions associated with childhood AIS include a great variety of diseases such as cardiac disease, hematological disorders, cerebral arteriopathies, trauma, infections, metabolic diseases, and collagen tissue abnormalities (1,6,7). In addition, hypercoagulable states associated with different inherited or acquired prothrombotic disorders are being increasingly recognized as possible risk factors for AIS (8).

The most studied prothrombotic risk factors include deficiencies of natural coagulation inhibitors such as antithrombin III, protein C, and free protein S, and genetic polymorphisms encoding proteins that constitute the coagulation system: factor V Leiden (FVL) and factor II 20210A (FII 20210A). Furthermore, hyperhomocysteinemia, elevated lipoprotein (a) values, and the presence of antiphospholipid antibodies (APA) have been reported as additional prothrombotic risk factors (8-13). Although individual prothrombotic risk factors are less important risk factors for childhood stroke, the presence of multiple prothrombotic risk factors may increase its risk (1,9).

The distribution of prothrombotic risk factors may vary among different age groups, stroke subtypes, and different populations (14). The aim of the present study was to determine the frequency of common inherited and acquired prothrombotic risk factors in Croatian children with an established diagnosis of AIS and TIA of undetermined etiology, and to identify possible cases of presence of multiple risk factors in children with AIS and TIA.

## Patients/material and methods

This research was performed as a part of a large clinical observation study (the project approved by the Ministry of Science of Croatia) on the role and prevalence of prothrombotic risk factors in children with cerebrovascular events.

### Participants

From September 2000 to June 2007, 161 children with symptoms of focal neurological deficit were admitted to the Department of Neuropediatrics at the Children’s Hospital Zagreb. Also, 18 asymptomatic children were referred from primary care health center due to medical history of suspected TIA. Asymptomatic patients were admitted to the hospital within 48 hours of the onset of symptoms indicating an acute cerebrovascular event. Children who showed no symptoms at the admission were diagnosed with TIA and kept at the Neuropediatric Department for observation in the same way as the children admitted with the symptoms.

**Figure 1:**
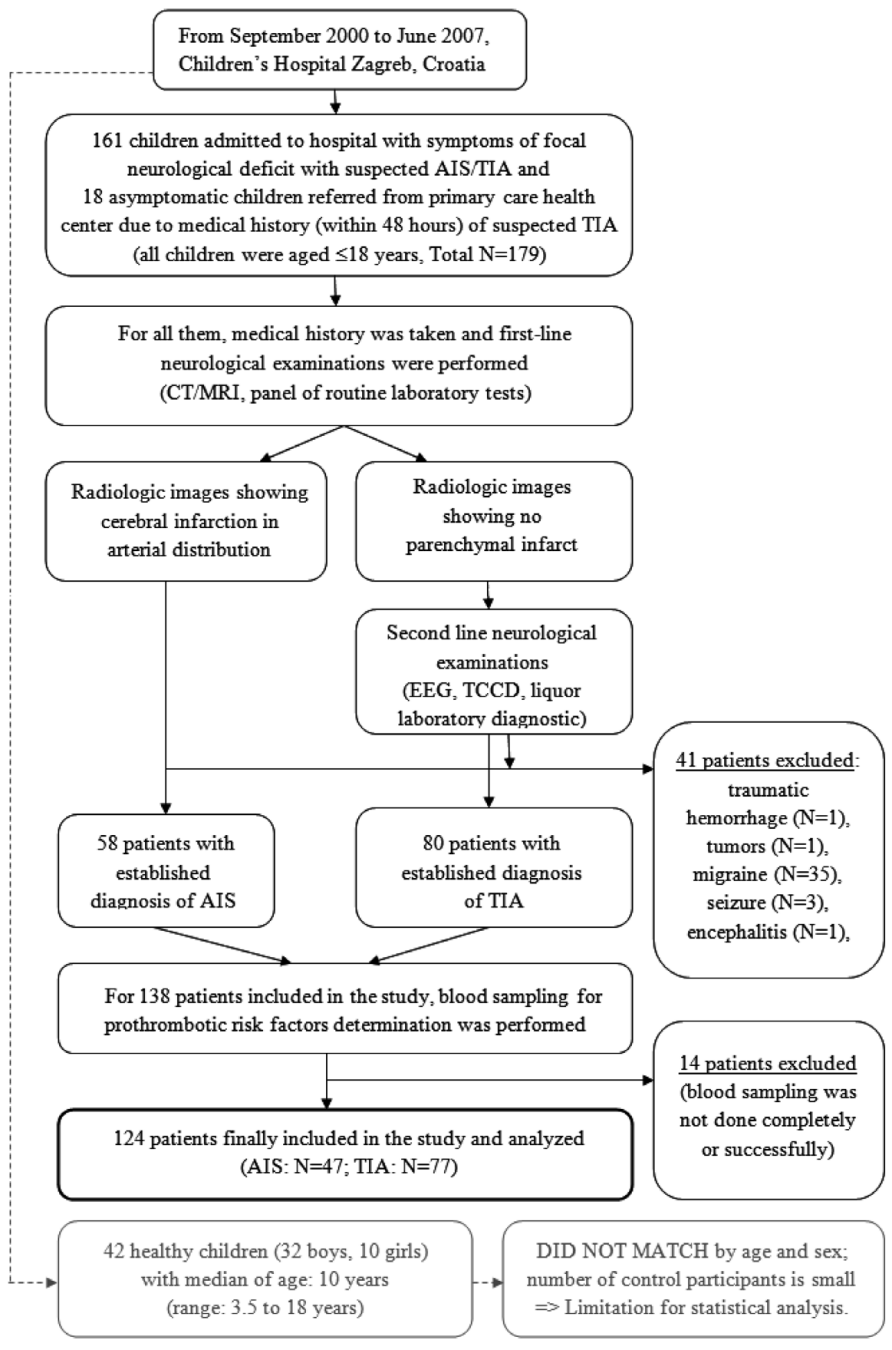
Study population, inclusion/exclusion criteria, and participants enrolled in the study. AIS – arterial ischemic stroke; TIA – transient ischemic attacks; CT – computed tomography; MRI – magnetic resonance imaging; EEG – electroencephalography; TCCD – transcranial color Doppler ultrasonography.

Of the initial 179 children, aged ≤18 years, from different regions of Croatia, 55 children (30.7%) were excluded (Figure 1). In 41 children, the diagnosis of AIS or TIA was not confirmed and in 14 children blood sampling was not done successfully or completely. Finally, 124 children were included in the study (47 children with AIS and 77 children with TIA). The control group consisted of 42 children (32 boys, 10 girls) aged ≤18 years from the same region with no history of neurologic or thromboembolic diseases, who were recruited among children waiting a minor surgery (adenotonsillectomy). The informed consent was obtained from the parents, and the study was approved by the ethics committee of Children’s Hospital Zagreb.

### Inclusion/exclusion criteria

During the hospital stay, the diagnosis was established on the basis of careful clinical history-taking and physical and neurological examinations, and documented with at least one brain imaging technique, computed tomography (CT) or magnetic resonance imaging (MRI). Furthermore, electroencephalographic (EEG) examinations, transcranial color Doppler ultrasonography (TCCD), and a panel of routine laboratory tests (complete blood count, erythrocyte sedimentation rate, C-reactive protein, global coagulation test, acid-base status, global biochemistry panel, and routine urine examination) were performed.

Inclusion criteria for this study were clinical symptoms of AIS/TIA in children: hemiparesis or monoparesis, seizures, headache, spasticity, hypotonia, vomiting, aphasia, vertigo, and/or ataxia. Brain imaging technique (CT and/or MRI) was performed in all patients. If radiologic evidence of cerebral infarction in arterial distribution was present, the diagnosis of AIS was established. Otherwise, detailed examination (EEG, TCCD, routine laboratory tests) was performed to rule out migraine, seizure, encephalitis, traumatic hemorrhage, and/or tumor, thus establishing the diagnosis of TIA.

Control participants were children from the same geographical region as patients, hospitalized during the same period. All children underwent routine preoperative examination that included radiological imaging of the heart and lungs, electrocardiography, as well as a panel of routine laboratory tests (complete blood count, erythrocyte sedimentation rate, global coagulation test, acid-base status, global biochemistry panel, and routine urine examination) and examination by anesthesiologist. The study included only healthy children, who received the approval of an anesthesiologist. Exclusion criteria were neurological or thrombotic disease/risk factors (Figure 1).

### Blood samples

Blood samples were collected from patients within two days of the acute ischemic cerebrovascular event. At the time of blood sampling, the children were medication-free. Blood samples of control participants were collected at the same time when venepunction was performed for preoperative blood tests examination. Blood samples of all participants were taken according to good laboratory practice (15).

Blood for coagulation analysis was drawn into Vacutainer tubes containing 0.109 M buffered sodium citrate, and centrifuged within 30 minutes twice at 2000 g for 15 minutes at room temperature. Functional activities of antithrombin III and protein C were tested immediately, whereas plasma sample aliquots for the determination of free protein S antigen and the presence of lupus anticoagulant (LA) were stored at -35°C, and assayed in batches. In cases when results of laboratory tests PC and free PS were pathological (according to age-specific reference interval), blood sampling to determine these parameters was repeated after three to six months at the regular neuropediatric examination (16).

For the determination of fasting homocysteine (Hcy), blood was collected into tubes containing K_3_-EDTA, placed immediately on ice, and centrifuged within 30 minutes at 1500 g for 5 minutes. Plasma samples were stored at -20°C until assaying.

Blood for serum samples was drawn into Vacutainer tubes without additives. Serum samples for the determination of Lp (a), triglycerides, total cholesterol, high- and low-density lipoprotein (HDL- and LDL-cholesterol), anticardiolipin (aCA), and antiphosphatidyl-serine (aPS) antibodies were aliquoted and stored at -20°C until assaying. For genetic analysis, EDTA whole blood samples were frozen at -20°C until DNA extraction.

### Assays

Functional activities of antithrombin III and protein C, using chromogenic assays, were measured on a Sysmex CA-500 coagulation analyzer (Siemens Medical Solutions Diagnostics, IL, Deerfield, USA). Free protein S antigen was determined by enzyme-linked immunosorbent assay (ELISA) according to the method of Comp et al (17). The presence of LA was determined according to the criteria of the Scientific and Standardization Committee of the International Society on Thrombosis and Hemostasis (18). Determination of aCA and aPS (IgA, IgG, and IgM isotypes) was performed by using commercially available ELISA assays (Euroimmun AG, Lübeck, Germany).

Fasting total plasma Hcy was measured using the fluorescence polarization immunoassay method on the IMx analyzer (Abbott Diagnostics, Abbott Park, IL, USA). Total cholesterol and triglycerides were measured using standard enzymatic methods, while HDL-cholesterol was determined using a homogenous direct method (Olympus Diagnostics, GmbH, Hamburg, Germany). LDL-cholesterol levels were calculated using the Friedewald equation, whereas lipoprotein (a) concentrations were measured using the immunoturbidimetric method (Roche Diagnostics, Basel, Switzerland).

Results of coagulation analyses, tHcy, triglycerides, total cholesterol, HDL- and LDL-cholesterol were classified according to age-specific reference intervals (19-23). Serum concentrations of lipoprotein (a)>0.3 g/L were regarded as elevated, according to previously identified threshold value for venous thrombosis in childhood and increased cerebrovascular and cardiovascular risk in adults (24,25). For all aCA and aPS isotypes, cut-off values were calculated and considered as positive for value over the 99th percentile for normal subjects (26).

Genomic DNA was extracted according to standard procedures using the salting-out method (27). Factor V Leiden and the FII 20210A were determined by polymerase chain reaction-restriction fragment length polymorphism (PCR-RFLP) method. A 287-bp fragment encompassing nucleotide position 1691 of factor V gene was amplified with primers, according to Zöller et al (28). After the digestion with *Mnl*I (Stratagene, Austin, TX, USA), the wild type allele (1691G allele) resulted in 37-bp, 93-bp, and 157-bp fragments, whereas the mutant allele (1691A allele) resulted in 130-bp and 157-bp fragments. Analysis for FII 20210A was performed according to the method described by Poort et al (29). After the digestion of amplified 345-bp fragments with *Hind* III (Roche Diagnostics, Mannheim, Germany), the mutant A allele was cleaved in two 23-bp and 322-bp fragments, whereas the wild type G allele remained undigested. Digested PCR products were separated by electrophoresis on 1.5% agarose gels (Applied Biosystems, Foster City, CA, USA) for factor V Leiden and on Spreadex gels (Guest Elchrom Scientific, Cham, Switzerland) for FII 20210A.

All measured prothrombotic risk factors were classified into five groups: natural coagulation inhibitors (antithrombin III, protein C, free protein S antigen), blood coagulation factors mutations (FVL and FII 20210A), homocysteine, lipid and lipoprotein profile (lipoprotein (a), triglycerides, total cholesterol, HDL- and LDL-cholesterol), and APA group factors (LA, aCA, and aPS).

### Presentation of results

Results are presented as relative frequencies of all observed variables (proportion and percentage). Pathological values represent the values outside of the applied reference intervals (by age and sex) or cut-off values (Table 1). Pathological value of polymorphisms FVL and F II 20210 was the “dominant” model (homozygous or heterozygous variant in comparison to the homozygous wild-type). Statistical analysis of the results was not performed because control group did not match by age and sex.

**Table 1:** Applied reference intervals (by age and sex) or used cut-off values of studied risk factors

Age	Limit
**Antithrombin III** (% activity)	
<6 d	<51.0
<3 mo	<54.0
<6 mo	<63.0
>6 mo and adults	<75.0
**Protein C** (% activity)	
<6 d	<26.0
<3 mo	<41.0
<6 mo	<48.0
>6 mo and adults	<75.0
**Protein S, free** (% activity)	
<6 d	<24.0
<3 mo	<40.0
<6 mo	<44.0
>6 mo and adults	<70.0
**Homocysteine (**µmol/L)	
<12 y	>7.6
>12 y	>15.0
**Cholesterol, total** (mmol/L)	
<1 y	>4.7
>1 y	>5.0
**Cholesterol, high density lipoprotein** (mmol/L)	
<18 y	<0.9
**Cholesterol, low density lipoprotein** (mmol/L)	
<18 y	>4.3
**Triglycerides** (mmol/L)	
<1 y	>2.3
>1 y	>1.7
**Lipoprotein (a)** (g/L)	
<18 y	>0.3
**Anticardiolipin antibodies** (PL-U/mL)	
<18 y	>25
**Antiphosphatidyl-serine antibodies** (RU-U/mL)	
<18 y	>40
**Lupus anticoagulant (**ratio)	
<18 y	>1.4
Polymorphisms **FVL** and **F II 20210A**	
<18 y	AA, GA:GG*

*“Dominant” model: homozygous (AA) or heterozygous (GA) variant in comparison with the homozygous wild-type (GG).

## Results

Out of 124 children, the diagnosis of AIS was established in 47 children (30 boys, 17 girls), median age 7 years (6 months-18 years). The remaining 77 children (33 boys, 44 girls) were diagnosed with TIA (median age 12 years) (3-18). The control group included 42 healthy children (32 boys and 10 girls with median age of 10 years and range from 3.5 to 18 years).

Patients and controls most frequently had pathological values of the prothrombotic risk factors in the lipid and lipoprotein profile group, followed by the natural coagulation inhibitors, tHcy, blood coagulation factor polymorphisms, and APA group (Table 2).

**Table 2:** Distribution of prothrombotic risk factors in patients and control group. Obtained values are given as counts and percentages for each risk factor and total for a group of risk factors

Risk factor	Arterial ischemic stroke (N = 47)	Transient ischemic attack (N = 77)	Controls (N = 42)
**Natural coagulation inhibitors**			
**Antithrombin III (%)**	3 (6.4)	0 (0.0)	0 (0.0)
**Protein C (%)**	6 (12.8)	8 (10.4)	0 (0.0)
**Free protein S antigen** **(%)**	3 (6.4)	9 (11.7)	0 (0.0)
Total of factors	12	17	0
**Number of patients with**			
0 factors	39 (83.0)	62 (80.5)	42(100.0)
1 factor	6 (12.8)	13 (16.9)	0 (0.0)
2 factors	3 (6.4)	2 (2.6)	0 (0.0)
Total of patients with any factor	9 (19.1)	15 (19.5)	0 (0.0)
**Blood coagulation factors**			
**FV Leiden**			
GA heterozygote	2 (4.3)	3 (3.9)	2 (4.8)
AA homozygote	0 (0.0)	0 (0.0)	0 (0.0)
**FII 20210A**			
GA heterozygote	2 (4.3)	2 (2.6)	1 (2.4)
AA homozygote	0 (0.0)	0 (0.0)	0 (0.0)
Total of factors	4	5	3
**Number of patients with**			
0 factors	44 (93.6)	72 (93.5)	39 (92.9)
1 factor	2 (4.3)	5 (6.5)	3 (7.1)
2 factors	1 (2.1)	0 (0.0)	0 (0.0)
Total of patients with any factor	3 (6.4)	5 (6.5)	3 (7.1)
**Homocysteine (μmol/L)**	7 (14.9)	9 (11.7)	5 (11.9)
**Lipid and lipoprotein profile**	15 (31.9)	24 (31.2)	10 (23.8)
Triglycerides (mmol/L)	5 (10.6)	2 (2.6)	1 (2.4)
Total cholesterol (mmol/L)	3 (6.4)	0 (0.0)	1 (2.4)
High density lipoprotein-cholesterol (mmol/L)	4 (8.5)	7 (9.1)	2 (4.8)
Low density lipoprotein-cholesterol (mmol/L)	5 (10.6)	1 (1.3)	2 (4.8)
Total of factors	32	34	16
**Number of patients with**			
0 factors	26 (55.3)	47 (61.0)	29 (69.0)
1 factor	12 (25.5)	27 (35.0)	11 (26.2)
2 factors	7 (14.9)	2 (2.6)	1 (2.4)
3 factors	2 (4.3)	1 (1.3)	1 (2.4)
Total of patients with any factor	21(44.7)	30 (39.0)	13 (31.0)
**Antiphospholipid antibodies**			
**Anticardiolipin antibodies**	0 (0.0)	1 (1.3)	3 (7.1)
IgG	0	1	1
IgA	0	0	1
IgM	0	0	1
**Antiphosphatidyl-serine antibodies**	4 (8.5)	0 (0.0)	1 (2.4)
IgG	4	0	0
IgA	0	0	0
IgM	0	0	0
Lupus anticoagulant	0 (0.0)	0 (0.0)	(0.0)
Total of factors	4	1	4
Number of patients with:			
0 factors	43 (91.5)	76 (98.7)	38 (80.9)
1 factor	4 (8.5)	1 (1.3)	2 (4.8)
2 factors	0 (0.0)	0 (0.0)	1 (2.4)
Total of patients with any factor	4 (8.5)	1 (1.3)	3 (7.1)

We found 32 pathological values in the lipids and lipoproteins group in 21/47 children with AIS (44.7%), and 2 or 3 values in 9 children (Table 2). Similarly, in children with TIA we found 34 pathological values in the lipids and lipoproteins group in 30/77 children (39.0%) and 2 or 3 values in 3 children. Two out of 13/42 (31.0%) healthy children showed the presence of multiple pathological values of lipid/lipoproteins.

In this group of risk factors, the risk factor with most frequent pathological values was lipoprotein (a), which usually occurred in combination with elevated LDL-cholesterol (3/5) and elevated triglycerides (4/5) in children with AIS. In children with TIA and the control group, elevated levels of lipoprotein (a) were mostly an independent factor and were found in combination with lower HDL-cholesterol and elevated LDL-cholesterol levels only in individual cases.

In the group of natural coagulation inhibitors**,** pathological values were found in 9/47 (19.1%) of children with AIS, 15/77 (19.5%) children with TIA and in no children in the control group. In most cases, decreased value of antithrombin III, protein C, and free protein S were independent factors. Two patients with AIS and TIA had a decreased value of protein C and protein S at the same time, and only one child with AIS had a decreased value of PC and antithrombin III.

Elevated homocysteine concentrations, the presence of FVL and FII 20210 polymorphisms as well as the presence of antiphospholipid antibodies were equally represented in all groups. All children with FVL and FII 20210A were heterozygous and one child in the AIS group had both polymorphisms. The overall representation of risk factors from the APA group was small. In all patients, LA was negative.

**Figure 2:**
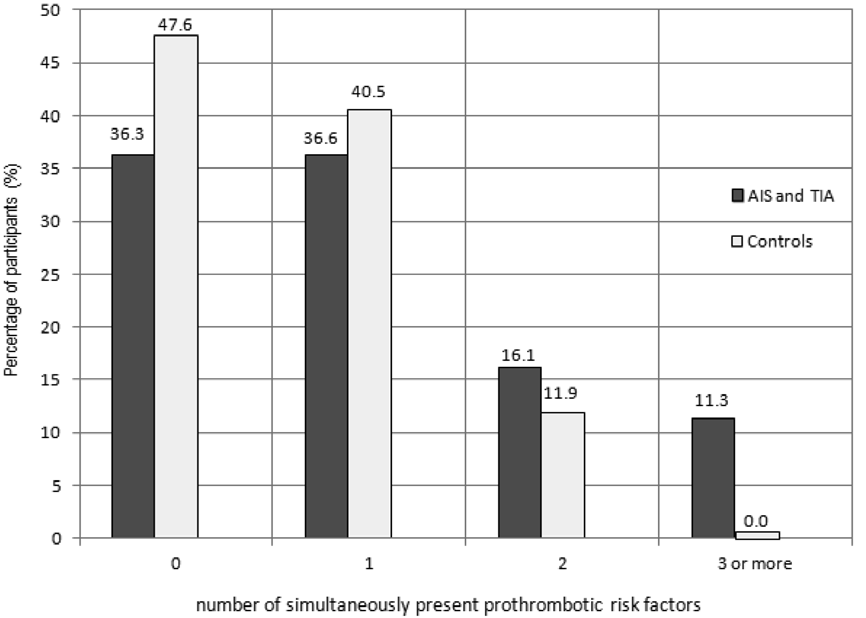
The percentage of participants with simultaneously present prothrombotic risk factors. In the control group there were no cases with three or more simultaneously present prothrombotic risk factors. AIS – arterial ischemic stroke; TIA – transient ischemic attacks.

Forty-five out of 124 (36.3%) children with AIS/TIA and 20/42 (47.6%) controls did not have any prothrombotic risk factor. The presence of one or two risk factors at the same time was found in all groups, but the presence of three or more at same time was found only in children with AIS and TIA (14/124; 11.3%).(Figure 2)

## Discussion

The most common prothrombotic risk factor in our study was elevated lipoprotein (a). About one third of children with AIS and TIA and about fifth of controls had pathological values of lipoprotein (a). Previously published childhood stroke reports found elevated values of lipoprotein (a) in between 15% and 29% of examinees (10,14,30-32). The same studies reported a lower proportion of elevated lipoprotein (a) in the control group than in our study. The reason for this may be non-standardized determination methods and reference values (33).

Low-activity natural coagulation inhibitors in our study had frequencies from 6.4% to 12.8%, with the lowest frequency of antithrombin III and approximately the same frequency of protein C and free protein S in children with AIS and TIA. In the control group, there were no cases with low activities of natural coagulation inhibitors. Such results are in accordance with previously published data (10,13,14,31,32,34). These studies suggest that only protein C can be used as a risk factor for childhood AIS. However, our results show that both protein C and protein S could be prothrombotic risk factors for cerebrovascular events (AIS and TIA).

Frequencies of FVL and FII 20210 in our study were lower than some previously published (31,33), but in the same range as those found in a recent meta-analysis (13,14) and similar to those recently published in the neighboring region (35,36).

To our knowledge, this is the first study in children with AIS that included aPS in the prothrombotic workup, besides the two most commonly tested APAs, LA and aCA. Although the presence of APA (especially aCA) has been reported to be associated with a 6-fold risk of stroke in children (11) and aPS has been reported to be a new prothrombotic risk factor and involved in the etiology of AIS (37-39), the number of positive aCA and aPS in our study was small and LA was negative in all participants.

Our study showed that multiple investigated risk factors could be a common hallmark of cerebrovascular disorders in children. The presence of one or two risk factors in the same child was found in all investigated groups, but the simultaneous presence of three or more factors was found only among children with AIS and TIA. This finding cannot be compared with previous findings for three reasons: 1) meta-analyses provide information on just two or more simultaneous risk factors (10,13,14,30) and some of them include other known risk factors, not only prothrombotic ones (40); 2) there are individual studies involving specific and smaller panels of risk factors (9,35); and 3) there are no published data about the frequency of prothrombotic risk factors in children with TIA, except in one study with only inherited prothrombotic risk factors (36).

An important limitation of this study is the control group because the number of healthy children was small and they were not matched with patients according to age and sex. The reason for this was mostly the need to reduce blood sampling since frequent blood sampling in preschool children can lead to anemia and very rare adenotonsillectomies under 3 years of age.

In conclusion, the presence of multiple prothrombotic risk factors in children with cerebrovascular disorder suggests that a combination of risk factors rather than individual risk factors could contribute to cerebrovascular disorders in children (AIS/TIA).

## References

[1] Amlie-Lefond C, Sebire G, Fullerton HJ (2008). Recent developments in childhood arterial ischemic stroke.. Lancet Neurol.

[2] deVeber G (2002). Stroke and the child’s brain: an overview of epidemiology, syndromes and risk factors.. Curr Opin Neurol.

[3] Agrawal N, Johnston SC, Wu YW, Sidney S, Fullerton HJ (2009). Imaging data reveal a higher pediatric stroke incidence than prior US estimates.. Stroke.

[4] Nelson KB, Lynch JK (2004). Stroke in newborn infants.. Lancet Neurol.

[5] Lenicek Krleza J, Đuranovic V, Lujic L, Coen Herak D, Mejaski-Bosnjak V, Nakic M (2009). The burden of pediatrics stroke and cerebrovascular disorders in Croatia.. Int J Stroke.

[6] Roach ES, Golomb MR, Adams R,, Biller J, Daniels S, Deveber G (2008). Management of stroke in infants and children: a scientific statement from a Special Writing Group of the American Heart Association Stroke Council and the Council on Cardiovascular Disease in the Young.. Stroke.

[7] Kirkham F, Sebire G, Steinlin M, Sträter R (2004). Arterial ischemic stroke in children.. Thromb Haemost.

[8] Barnes C, deVeber G (2006). Prothrombotic abnormalities in childhood ischemic stroke.. Thromb Res.

[9] Nestoridi E, Buonanno FS, Jones RM, Krishnamoorthy K, Grant PE, Van Cott EM (2002). Arterial ischemic stroke in childhood: the role of plasma-phase risk factors.. Curr Opin Neurol.

[10] Nowak-Gottl U, Strater R, Heinecke A, Junker R, Koch HG, Schuierer G (1999). Lipoprotein (a) and genetic polymorphisms of clotting factor V, prothrombin, and methylenetetrahydrofolate reductase are risk factors of spontaneous ischemic stroke in childhood.. Blood.

[11] Kenet G, Sadetzki S, Murad H, Martinowitz U, Rosenberg N, Gitel S (2000). Factor V Leiden and antiphospholipid antibodies are significant risk factors for ischemic stroke in children.. Stroke.

[12] Van Beynum IM, Smeitink JA, den Heijer M, te Poele Pothoff MT, Blom HJ (1999). Hyperhomocysteinemia: a risk factor for ischemic stroke in children.. Circulation.

[13] Kenet G Lutkhoff LK Albisetti M Bernard T Bonduel M Brandao L Impact of thrombophilia on risk of arterial ischemic stroke or cerebral sinovenous thrombosis in neonates and children: a systematic review and meta-analysis of observational studies. Circulation 2010 121 1838 47 Medline:20385928 10.1161/CIRCULATIONAHA.109.913673 20385928

[14] Lynch JK, Han CJ, Nee LE, Nelson KB (2005). Prothrombotic factors in children with stroke or porencephaly.. Pediatrics.

[15] Standards of good laboratory practice. Available from: http://www.hkmb.hr/povjerenstva/strucna-pitanja.html Accessed: July 31, 2013.

[16] Minuk L, Lazo-Langner A, Kovacs J, Robbins M, Morrow B, Kovacs M (2010). Normal levels of protein C and protein S tested in the acute phase of a venous thromboembolic event are not falsely elevated.. Thromb J.

[17] Comp PC, Doray D, Patton D, Esmon CT (1986). An abnormal plasma distribution of protein S occurs in functional protein S deficiency.. Blood.

[18] Brandt JT, Triplett DA, Alving B, Scharrer I (1995). Criteria for the diagnosis of lupus anticoagulants: an update.. Thromb Haemost.

[19] Andrew M, Paes B, Milner R, Johnston M, Mitchell L, Tollefsen DM (1987). Development of the human coagulation system in the full-term infant.. Blood.

[20] Andrew M, Vegh P, Johnston M, Bowker J, Ofosu F, Mitchell L (1992). Maturation of the hemostatic system during childhood.. Blood.

[21] Ehrenforth S, Junker R, Koch HG, Kreuz W, Münchow N, Scharrer I (1999). Multicenter evaluation of combined prothrombotic defects associated with thrombophilia in childhood.. Eur J Pediatr.

[22] Tonstad S, Refsum H, Silversten M, Christophersen B, Ose L, Ueland PM (1996). Relation of total homocysteine and lipid levels in children to premature cardiovascular death in male relatives.. Pediatr Res.

[23] (1992). National Cholesterol Education Program Report of the Expert Panel on Blood Cholesterol Levels in Children and Adolescents. Pediatrics.

[24] Nowak-Gottl U, Junker R, Hartmeier M, Koch HG, Münchow N, Assmann G (1999). Increased lipoprotein (a) is an important risk factor for venous thromboembolism in childhood.. Circulation.

[25] Rader DJ, Hoeg JM, Brewer HB (1994). Quantification of plasma apolipoproteins in the primary and secondary prevention of coronary artery disease.. Ann Intern Med.

[26] Tincani A, Allegri F, Balestrieri G, Reber G, Sanmarco M, Meroni P (2004). Minimal requirements for antiphospholipid antibodies ELISAs proposed by the European Forum on antiphospholipid antibodies.. Thromb Res.

[27] Miller SA, Dykes DD, Polesky HF (1988). A simple salting out procedure for extracting DNA from human nucleated cells.. Nucleic Acids Res.

[28] Zoller B, Svensson PJ, He X, Dahlback B (1994). Identification of the same factor V gene mutation in 47 out of 50 thrombosis-prone families with inherited resistance to activated protein C.. J Clin Invest.

[29] Poort SR, Rosendaal FR, Reitsma PH, Bertina RM (1996). A common genetic variation in the 3′-untranslated region of the prothrombin gene is associated with elevated plasma prothrombin levels and an increase in venous thrombosis.. Blood.

[30] Ganesan V, Prengler M, Mc Shane M, Wade AM, Kirkham FJ (2003). Investigation of risk factors in children with arterial ischemic stroke.. Ann Neurol.

[31] Haywood S, Liesner R, Pindora S, Ganesan V (2005). Thrombophilia and first arterial ischaemic stroke: a systematic review.. Arch Dis Child.

[32] Strater R, Vielhaber H, Kassenbohmer R, von Kries R, Gobel U, Nowak-Gottl U (1999). Genetic risk factors of thrombophilia in ischaemic childhood stroke of cardiac origin: a prospective ESPED survey.. Eur J Pediatr.

[33] Langer C, Tambyrayah B, Thedieck S, Nowak-Gottl U (2011). Testing for lipoprotein(a) concentration and apolipoprotein(a) phenotypes: method standardization and pediatric reference values.. Semin Thromb Hemost.

[34] Kenet G, Sadetzki S, Murad H, Martinowitz U, Rosenberg N, Gitel S (2000). Factor V Leiden and antiphospholipid antibodies are significant risk factors for ischemic stroke in children.. Stroke.

[35] Djordjevic V, Stankovic M, Brankovic-Sreckovic V, Rakicevic L, Damnjanovic T, Antonijevic N (2012). Prothrombotic genetic risk factors in stroke: a possible different role in pediatric and adult patients.. Clin Appl Thromb Hemost.

[36] Coen Herak D, Radic Antolic M, Lenicek Krleza J, Pavić M, Dodig S, Duranovic V (2009). Inherited prothrombotic risk factors in children with stroke, transient ischemic attack, or migraine.. Pediatrics.

[37] Kahles T, Humpich M, Steinmetz H, Sitzer M, Lindhoff-Last E (2005). Phosphatidylserine IgG and beta-2-glycoprotein I IgA antibodies may be a risk factor for ischaemic stroke.. Rheumatology.

[38] Toschi V, Motta A, Castelli C, Paracchini ML, Zerbi D, Gibelli A (1998). High prevalence of antiphosphatidylinositol antibodies in young patients with cerebral ischemia of undetermined cause.. Stroke.

[39] Tuhrim S, Rand JH, Wu X, Horowitz DR, Weinberger J, Goldman ME (1999). Antiphosphatidyl serine antibodies are independently associated with ischemic stroke.. Neurology.

[40] Mackay MT, Wiznitzer M, Benedict SL, Lee KJ, deVeber GA, Ganesan V (2011). at al. Arterial ischemic stroke risk factors: the International Pediatric Stroke Study.. Ann Neurol.

